# Serum neurofilament light chain levels associations with gray matter pathology: a 5‐year longitudinal study

**DOI:** 10.1002/acn3.50872

**Published:** 2019-08-22

**Authors:** Dejan Jakimovski, Jens Kuhle, Murali Ramanathan, Christian Barro, Davorka Tomic, Jesper Hagemeier, Harald Kropshofer, Niels Bergsland, David Leppert, Michael G. Dwyer, Zuzanna Michalak, Ralph H. B. Benedict, Bianca Weinstock‐Guttman, Robert Zivadinov

**Affiliations:** ^1^ Buffalo Neuroimaging Analysis Center, Department of Neurology, Jacobs School of Medicine and Biomedical Sciences University at Buffalo, State University of New York Buffalo New York; ^2^ Neurologic Clinic and Policlinic, Departments of Medicine, Biomedicine and Clinical Research University Hospital Basel, University of Basel Basel Switzerland; ^3^ Department of Pharmaceutical Sciences University at Buffalo, State University of New York Buffalo New York; ^4^ Novartis Pharma AG Basel Switzerland; ^5^ Center for Biomedical Imaging at Clinical Translational Science Institute University at Buffalo, State University of New York Buffalo New York; ^6^ Jacobs MS Center, Department of Neurology, Jacobs School of Medicine and Biomedical Sciences University at Buffalo, State University of New York Buffalo New York

## Abstract

**Background:**

Gray matter (GM) pathology is closely associated with physical and cognitive impairment in persons with multiple sclerosis (PwMS). Similarly, serum neurofilament light chain (sNfL) levels are related to MS disease activity and progression.

**Objectives:**

To assess the cross–sectional and longitudinal associations between sNfL and MRI–derived lesion and brain volume outcomes in PwMS and age–matched healthy controls (HCs).

**Materials and Methods:**

Forty‐seven HCs and 120 PwMS were followed over 5 years. All subjects underwent baseline and follow–up 3T MRI and sNfL examinations. Lesion volumes (LV) and global, tissue–specific and regional brain volumes were assessed. sNfL levels were analyzed using single molecule array (Simoa) assay and quantified in pg/mL. The associations between sNfL levels and MRI outcomes were investigated using regression analyses adjusted for age, sex, baseline disease modifying treatment (DMT) use and change in DMT over the follow‐up. False discovery rate (FDR)–adjusted *q*‐values <0.05 were considered significant.

**Results:**

In PwMS, baseline sNfL was associated with baseline T_1_‐, T_2_‐ and gadolinium‐LV (*q* = 0.002, *q* = 0.001 and *q* < 0.001, respectively), but not with their longitudinal changes. Higher baseline sNfL levels were associated with lower baseline deep GM (*β* = −0.257, *q* = 0.017), thalamus (*β* = −0.216, *q* = 0.0017), caudate (*β* = −0.263, *q* = 0.014) and hippocampus (*β* = −0.267, *q* = 0.015) volumes. Baseline sNfL was associated with longitudinal decline of deep GM (*β* = −0.386, *q* < 0.001), putamen (*β* = −0.395, *q* < 0.001), whole brain (*β* = −0.356, *q* = 0.002), thalamus (*β* = −0.272, *q* = 0.049), globus pallidus (*β* = −0.284, *q* = 0.017), and GM (*β* = −0.264, *q* = 0.042) volumes. No associations between sNfL and MRI–derived measures were seen in the HCs.

**Conclusion:**

Higher sNfL levels were associated with baseline LVs and greater development of GM atrophy in PwMS.

## Introduction

Multiple sclerosis (MS) is a chronic, inflammatory, demyelinating disease of the central nervous system which presents with reoccurring and transient neurological deficits followed frequently by insidious accumulation of physical and cognitive disability.^1^ Furthermore, emerging evidence identify neurodegenerative processes as one of the major contributors to long–term MS disability accumulation.^2^ Therefore, there is an increasing need for establishing simple, easily accessible, and accurate biomarkers that are able to quantify neuro–axonal injury and neurodegeneration. As such, magnetic resonance imaging (MRI)–derived brain volume measures and serum– and cerebrospinal fluid (CSF)–derived neurodegenerative biomarkers have been extensively investigated.^3^, ^4^

Persistent inflammation, concurrent axonal transection, and increased oxidative stress lead to a common final pathway of neuronal apoptosis and initiation of Wallerian degeneration.^5^ Thus neurodegenerative processes are substantially greater in highly connected structures (i.e. thalamus) and their reciprocal cortical areas.^6^ Multiple studies have highlighted the importance of gray matter (GM) pathology as the main driver of MS disability.^7^, ^8^ A multicenter study demonstrated that atrophy of the highly connected deep GM (DGM) was the only GM–associated region associated with concurrent disability progression.^9^ Moreover, thalamus atrophy, the largest structure within the DGM, has been recognized as an important biomarker of disease progression that can be utilized consistently throughout the disease and regardless of disease phenotype.^10^, ^11^ Contrarily, recent evidence suggests that an independent and pial–driven neurodegenerative pathology contributes to generalized neuronal loss and subsequent atrophy of the neighboring cortex.^12^, ^13^

Neurofilament light chain (NfL) is the main scaffolding component of the axonal cytoskeleton. Multiple neurodegenerative diseases are highlighted by damage and loss of the neuro–axonal unit, processes which result in abnormally high NfL levels in CSF and in blood.^14^ Despite the relatively low serum concentrations of NfL, recent technological development of single molecule array (Simoa) assays allow for reliable quantification.^15^ Such analyses were previously utilized in MS cohorts and showed good predictive ability for concurrent and future MS disability, and of global MRI changes.^16^, ^17^

Based on this background, we aimed to analyze associations between serum neurofilament (sNfL) levels with current and future neurodegenerative pathology in a heterogeneous group of persons with MS (PwMS) and age–matched healthy controls (HCs) over 5 years. In particular, we attempted to determine specific associations of sNfL with MS lesions, global brain volumes, and development of distinct pathology in the DGM, cortical and leptomeningeal structures.

## Methods

### Study population

The study utilized PwMS and HCs that were initially enrolled in a larger prospective, case‐controlled study which examined the cardiovascular, environmental, and genetic factors in PwMS (CEG‐MS).^13^, ^18^ The PwMS inclusion criteria for this substudy were: (1) being diagnosed as clinically definite MS according to the 2010‐revised McDonald criteria,^19^ (2) baseline age of 18–75 years, (3) both baseline and follow–up MRI examination within 30 days of the clinical visit, (4) use of the same imaging 3T protocol at baseline and follow‐up, and (5) baseline and follow–up availability of serum samples within 30 days from MRI. PwMS were excluded from the study if they had (1) a clinically documented relapse or 2) steroid use within the 30 days of the study date, and 3) were pregnant or nursing mothers. On the other hand, the study inclusion criteria for the HCs were: (1) baseline age of 18–75 years, (2) not being diagnosed with current nor past neurological disease, (3) both baseline and follow–up MRI examination within 30 days of the clinical visit, (4) use of the same imaging 3T protocol at baseline and follow‐up, and (5) baseline and follow–up availability of serum samples within 30 days from MRI.

Both at baseline and follow‐up, the PwMS underwent a full neurological examination and Expanded Disability Status Scale (EDSS) scores were derived.^20^ The heterogeneous group of PwMS was further classified as either relapsing–remitting MS (RRMS) or progressive MS (PMS) patients. The use of MS–specific disease modifying treatment (DMT) at baseline and follow–up time points was determined during the clinical visit. Due to the large number of DMT combinations, the longitudinal change was coded as either PwMS DMT switchers (different DMT at baseline and follow–up time points) or as PwMS with stable DMT (remained on baseline DMT during the entire study period). The study was approved by the University at Buffalo Institutional Review Board (IRB) and participants signed a written consent form.

### MRI acquisition and analysis

The MRI examinations were performed on a 3T GE Signa Excite HD 12 Twin Speed 8‐channel scanner (General Electric, Milwaukee, WI, USA) with an 8‐channel head and neck (HDNV) coil. There were no major MRI hardware or software changes over the follow–up period. The specific MRI acquisition parameters are provided in the Data S1.

T_1_, T_2_, and gadolinium (Gd)–enhancing lesion masks were obtained using semiautomated contouring/thresholding technique, as described elsewhere.^21^ Lesion volumes (LV) were further quantified in milliliters (mL). Accrual of new/enlarging T2‐LV over the follow–up period was additionally calculated.^22^

Prior to the brain volume segmentation, the T_1_ hypointensities were filled to avoid tissue misclassification.^23^ Whole brain volume (WBV), white matter volume (WMV), gray matter volume (GMV) and cortical volume (CV) were obtained with SIENAX software (version 2.6, FMRIB, Oxford, UK).^24^ The longitudinal percentage change of the WBV and the WMV, GMV, CV were calculated using SIENA^24^ and SIENAX multi‐time point algorithms, respectively.^25^ Similarly, the cross–sectional and longitudinal changes of regional tissue–specific volumes of the total DGM and the specific nuclei (thalamus, caudate, globus pallidus, putamen and hippocampus) were derived using the FMRIB’s Integrated Registration and Segmentation Tool (FIRST) software (FMRIB, Oxford, UK).^26^ Both global and DGM volumes were normalized with the SIENAX–derived scaling factor and quantified in milliliters.

The presence of leptomeningeal contrast enhancement (LMCE) was defined as post‐contrast signal intensity within the subarachnoid space which was substantially higher when compared to the brain parenchyma.^27^ The 3D‐FLAIR images were examined with Java Image Manipulation Tool (JIM) (Version 6.0, http://www.xinapse.com) and LMCEs were evaluated, as previously reported.^13^

### sNfL levels analysis

The blood samples were collected at the time of the MRI examination and properly stored. Later on, the samples were sent to the University of Basel where the baseline and follow–up levels of the sNfL were derived using a validated single molecule array (Simoa) assay and quantified in pg/mL. The full description of Simoa assay is published elsewhere.^15^

### Statistical analysis

Statistical analyses were performed using SPSS version 25.0 (IBM, Armonk, NY). The normal distribution of the data was determined using the Kolmogorov–Smirnov test. The differences between PwMS and HCs in demographic, clinical, sNfL levels, and MRI–derived outcomes were compared using *χ* ^2^ test, Student’s *t*‐test, one‐way analysis of variance (for parametric variables), Mann–Whitney *U*‐test (for nonparametric variables), age–adjusted analysis of covariance (ANCOVA), and negative binomial regression accordingly. The associations between the sNfL levels and the MRI–derived lesion and brain volumes were analyzed using linear regression models where the MRI variables were set as dependent variables and the sNfL levels, age, sex, baseline DMT and change in DMT over the follow‐up were set as independent variables. Regression model–derived *R* ^2^, beta (B), standard error (SE), standardized *β*, and *P*‐values were derived. All regression–model metrics are fully reported in the Data S1. Due to the data skewness of the sNfL and lesion volumes, the variables were logarithmically transformed before being used in the models. Due to the nature of the % change in Gd–enhancing LV variable, the data were initially transformed with zero‐inflated model and Poisson loglinear statistical modeling was performed. The associations between sNfL levels and the presence of LMCE was determined using logistic regression models. False discovery rate (FDR) correction was performed using Benjamini‐Hochberg procedure and adjusted *P*‐values (hereafter presented as *q*‐values) were calculated. *Q*‐values <0.05 were considered statistically significant.

A secondary, post hoc analysis employed a baseline sNfL cut‐off of 30 pg/mL.^28^ The PwMS were divided based on sNfL levels of <30 or ≥30 pg/mL and compared with previously described methods. The MRI–derived variables were corrected by baseline demographic and clinical differences. Alternatively, we performed two additional cut‐off analyses where the PwMS were labeled with “elevated sNfL levels” based on population averages and were determined as either the 97.5th or 99th percentiles for the age–specific 45‐year‐old HC population.^16^ The second analysis individually classified each subject with “elevated sNfL levels” as determined by the age–specific 95th and 97.5th sNfL reference.^16^ Lastly, associations between sNfL levels and MRI‐derived volumetric measures were further analyzed by additional correction for baseline Gd‐LV, T1‐LV accrual, and accrual of new/enlarging T2‐LV. Post hoc analyses were considered significant at *P* < 0.05.

## Results

### Demographic and clinical characteristics

Detailed demographic and clinical characteristics of the study populations are shown in Table 1. There were no significant differences between the PwMS patients and HCs in their age (48.1 vs. 44.5 years, *t*‐test *P* = 0.148) and in sex ratio (F/M, 34/13 vs. 85/35, *χ* ^2^ *P* = 1.000). Within the MS phenotype, the PMS (five primary–progressive and 31 secondary–progressive MS patients) group was older and had longer disease duration when compared to the RRMS population (56.5 vs. 44.6 years, *t*‐test *P* < 0.001 and 22.6 vs. 13.4 years, *t*‐test *P* < 0.001). Furthermore, there were significant differences in the annualized relapse rate (ARR) but not in the absolute change of EDSS over the follow–up period (RRMS ARR 0.219 vs. PMS ARR 0.09, negative binomial regression *P* = 0.008 and RRMS EDSS change of 0.4 vs. PMS EDSS change of 0.3, *t*‐test *P* = 0.663). There were no differences in the follow–up period between the groups (5.5 years for HCs, and 5.5 years for PwMS, *t*‐test *P* = 0.821). The particular DMT use at baseline and change in DMT over the follow–up period are also shown in Table 1.

**Table 1:** Demographic and clinical characteristics of the study population.

Clinical and demographic characteristics	HCs (*n* = 47)	PwMS (*n* = 120)	RRMS (*n* = 84)	PMS (*n* = 36)	*P*‐value PwMS vs. HCs	*P*‐value RRMS vs. PMS
Female, *n* (%)	34 (72.3)	85 (70.8)	57 (67.9)	28 (77.8)	1.000	0.273
Age at baseline, mean (SD)	44.5 (15.6)	48.1 (11.2)	44.6 (10.9)	56.5 (6.3)	0.148	**<0.001**
Follow–up period, mean (SD)	5.5 (0.5)	5.5 (0.5)	5.5 (0.6)	5.5 (0.4)	0.821	0.863
Disease duration at baseline, mean (SD)	–	16.2 (10.3)	13.4 (8.9)	22.6 (10.6)	–	**<0.001**
EDSS at baseline, median (IQR)	–	2.5 (1.5–5.0)	2.0 (1.5–3.0)	5.0 (3.5–6.5)	–	**<0.001**
EDSS at follow‐up, median (IQR)	–	3.5 (2.0–6.0)	2.5 (1.5–3.63)	6.0 (3.8–6.5)	–	**<0.001**
EDSS absolute change, mean (SD)	–	0.4 (0.9)	0.4 (1.0)	0.3 (0.7)	–	0.663
Relapse rate, mean (SD)	–	0.181 (0.424)	0.219 (0.466)	0.090 (0.287)	–	0.008*
sNfL at baseline, median (IQR)	15.3 (8.3–23.1)	20.6 (13.8–31.1)	18.1 (12.8–26.7)	25.8 (19.7–40.6)	**0.002**	0.172
sNfL at follow‐up, median (IQR)	16.7 (7.9–23.8)	23.8 (16.2–32.3)	20.6 (14.3–27.0)	32.2 (24.5–47.3)	**0.002**	**0.028**
sNfL absolute change, median (IQR)	2.3 (−1.1–5.9)	2.1 (−2.8–7.9)	0.9 (−4.8–5.9)	6.7 (−0.7–14.6)	0.976	0.612
LMCE at follow‐up, *n* (%)	–	31 (35.2)	17 (29.3)	14 (46.7)	–	0.106
DMT use at baseline, *n* (%)						
Interferon‐*β*	–	43 (35.8%)	29 (34.5%)	14 (38.9%)	–	0.096
I Glatiramer acetate	–	29 (24.2%)	18 (21.4%)	11 (30.6%)		
I Natalizumab	–	18 (15%)	17 (20.2%)	1 (2.8%)		
I Off–label medications	–	2 (1.7%)	1 (1.2%)	1 (2.8%)		
I No DMT	–	28 (23.3%)	19 (22.6%)	9 (25%)		
I Switchers/remaining on same DMT, *n*	–	42/78	33/51	9/27	–	0.242

HCs, healthy controls; PwMS, persons with multiple sclerosis; RRMS, relapsing, remitting multiple sclerosis; PMS, progressive multiple sclerosis; EDSS, Expanded Disability Status Scale; sNfL, serum neurofilament light chain; LMCE, leptomeningeal contrast enhancement; DMT, disease modifying treatment; SD, standard deviation; IQR, interquartile range.

Off–label medications included mitoxantrone (1) and azathioprine (1).

3D‐FLAIR postcontrast imaging required for LMCE analysis was available in 88 MS patients (58 RRMS, 30 PMS).

Student’s *t*‐test, Mann–Whitney *U*‐test, negative binomial regression and *χ* ^2^ test were used appropriately.

The differences in sNfL levels at baseline and follow‐up were calculated with analysis of covariance (ANCOVA) adjusted for baseline age and utilized logarithmically transformed sNfL data. In bold are displayed significant *P*‐values.

* The differences in relapse rate was calculated using the exact count data and with negative binomial regression modeling.

### sNfL levels in the study groups

**Figure 1:**
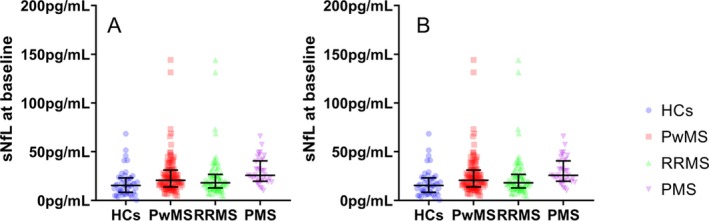
Box plot representation of baseline (A) and follow‐up (B) sNfL levels between the study populations. PwMS, persons with multiple sclerosis; HCs, healthy controls; RRMS, relapsing–remitting multiple sclerosis; PMS, progressive multiple sclerosis; sNfL, serum neurofilament light chain. Median and interquartile range are shown.

The differences in baseline, follow‐up, and longitudinal changes in sNfL levels between the HCs and the PwMS are shown in Table 1 and Figure 1. PwMS had higher median sNfL levels when compared to the HCs at both baseline and at 5‐year follow‐up (median 20.6 vs. 15.3 pg/mL, age–adjusted ANCOVA *q* = 0.002; and median 23.8 vs. 16.7 pg/mL, age–adjusted ANCOVA *q* = 0.002, respectively). Overall, there was no difference between the PwMS and HCs in the absolute sNfL change over the follow‐up (median 2.1 vs. 2.3 pg/mL, age–adjusted ANCOVA *q* = 0.976).

The baseline sNfL levels were not significantly higher in the PMS group when compared to the RRMS group (median 25.8 vs. 18.1 pg/mL, age–adjusted ANCOVA *q* = 0.172). On the other hand, the PMS group had higher sNfL at follow‐up (median 32.2 vs. 20.6 pg/mL, age–adjusted ANCOVA *q* = 0.028). The PMS group had numerically greater absolute longitudinal increase in sNfL levels, compared to the RRMS group (median 6.7 vs. 0.9, age–adjusted ANCOVA *q* = 0.612).

### MRI‐derived lesion and brain volume characteristics

The differences in cross–sectional and longitudinal changes of MRI–derived lesion and brain volumes are shown in Table 2. During the 5‐year follow‐up and compared to HCs, PwMS had lost significantly more total DGM volume (−5.9% vs. −3.9%, *t*‐test *P* = 0.003), caudate volume (−6.2% vs. −4.8%, *t*‐test *P* = 0.027) and hippocampus volume (−5.2% vs. −3.9%, *t*‐test *P* < 0.001).

**Table 2:** MRI–derived outcomes derived at baseline and over the follow‐up in HCs and PwMS.

MRI–derived volumes	HCs (*n* = 47)	PwMS (*n* = 120)	RRMS (*n* = 84)	PMS (*n* = 36)	HCs vs. PwMS *P*‐value	RRMS vs. PMS *P*‐value
Cross‐sectional at baseline						
T_1_‐LV, mean (SD)	–	3.5 (8.0)	3.1 (8.3)	4.4 (7.3)	**–**	**0.005**
T_2_‐LV, mean (SD)	0.4 (1.1)	15.9 (20.0)	13.5 (20.0)	21.2 (19.2)	**<0.001**	**0.001**
Gd‐LV, mean (SD)	–	0.1 (0.5)	0.1 (0.6)	0.005 (0.3)	–	0.102
Gd–positive scans, *n* (%)	–	12 (10)	11 (13,1)	1 (2.8)	–	0.084
WBV, mean (SD)	1531.1 (90.1)	1447.0 (100.7)	1473.5 (94.8)	1385.9 (87.1)	**<0.001**	**<0.001**
WMV, mean (SD)	749.4 (41.4)	718.5 (64.1)	732.4 (64.2)	686.4 (51.7)	**<0.001**	**<0.001**
GMV, mean (SD)	781.7 (60.7)	728.6 (63.6)	741.2 (66.2)	699.6 (45.8)	**<0.001**	**0.001**
CV, mean (SD)	637.1 (54.0)	590.6 (51.6)	600.4 (54.8)	567.9 (34.4)	**<0.001**	**<0.001**
DGM, mean (SD)	61.9 (4.3)	56.8 (7.3)	57.2 (7.5)	52.9 (5.9)	**<0.001**	**0.003**
Thalamus, mean (SD)	20.9 (1.8)	18.8 (2.7)	19.0 (2.6)	17.2 (2.2)	**<0.001**	**<0.001**
Caudate, mean (SD)	9.1 (1.3)	8.4 (1.3)	8.5 (1.4)	7.7 (1.0)	**<0.001**	**0.003**
Putamen, mean (SD)	13.1 (1.1)	12.1 (1.7)	12.1 (1.8)	11.3 (1.3)	**<0.001**	0.208
Globus pallidus, mean (SD)	4.7 (0.4)	4.3 (0.7)	4.3 (0.6)	4.2 (0.7)	**<0.001**	**0.046**
Hippocampus, mean (SD)	9.8 (1.0)	9.1 (1.3)	9.1 (1.4)	8.6 (1.0)	**<0.001**	0.573
Longitudinal change						
T_1_‐LV, mean (SD)	–	0.3 (1.8)	0.2 (1.9)	0.3 (1.7)	–	0.653
T_2_‐LV, mean (SD)	0.3 (0.9)	0.5 (5.2)	0.09 (5.7)	1.4 (3.3)	0.769	0.214
Gd‐LV, mean (SD)	–	−0.09 (0.6)	−0.1 (0.7)	−0.006 (0.03)	–	0.705
Gd‐positive scans, *n* (%)	–	5 (4.2)	4 (4.8)	1 (2.8)	–	0.618
WBV, mean (SD)	−2.9 (1.6)	−3.4 (2.1)	−3.6 (2.2)	−3.1 (1.8)	0.108	0.267
WMV, mean (SD)	−1.5 (2.6)	−1.5 (4.3)	−2.2 (4.9)	−0.1 (2.4)	0.976	**0.025**
GMV, mean (SD)	−2.5 (1.9)	−2.2 (2.8)	−1.9 (2.9)	−2.7 (2.1)	0.524	0.182
CV, mean (SD)	−2.9 (1.7)	−2.2 (3.0)	−2.0 (3.3)	−2.5 (2.4)	0.067	0.451
DGM, mean (SD)	−3.9 (3.9)	−5.9 (3.8)	−6.2 (4.3)	−5.5 (3.1)	**0.003**	0.365
Thalamus, mean (SD)	−1.3 (13.2)	−4.6 (7.2)	−6.7 (4.8)	−4.4 (3.1)	0.098	**0.002**
Caudate, mean (SD)	−4.8 (4.2)	−6.2 (4.3)	−4.8 (7.8)	−4.9 (5.8)	**0.027**	0.979
Putamen, mean (SD)	−4.3 (4.1)	−4.6 (6.9)	−4.5 (8.4)	−4.9 (4.4)	0.727	0.765
Globus pallidus, mean (SD)	−3.4 (4.9)	−9.2 (8.4)	−10.4 (9.7)	−8.8 (5.2)	0.163	0.333
Hippocampus, mean (SD)	−3.9 (5.3)	−5.2 (6.9)	−5.2 (6.7)	−6.0 (3.7)	**<0.001**	0.568

HCs, healthy controls; PwMS, persons with multiple sclerosis; RRMS, relapsing–remitting multiple sclerosis; PMS, progressive multiple sclerosis; LV, lesion volume; Gd, gadolinium; WBV, whole brain volume; WMV, white matter volume; GMV, gray matter volume; CV, cortical volume; DGM, deep gray matter; SD, standard deviation.

All brain volumes are shown in milliliters and are normalized using SIENAX–derived scaling factor. Longitudinal change for MRI–lesion derived outcomes is shown in mL, whereas for brain volumes, the percentage changes are displayed.

Student’s *t*‐test and Mann–Whitney *U*‐test were used and *P*‐values <0.05 were considered significant and displayed in bold.

When compared to RRMS, the PMS group had greater T_1_‐ and T_2_‐LV, lower global brain volumes, lower total DGM, thalamus, and caudate and volume at baseline (all *P* < 0.01). Over the follow–up period, the RRMS group lost significantly more WMV and thalamic volume when compared to PMS (−2.2% vs. −0.1%, *t*‐test *P* = 0.025 and −6.7% vs. −4.4%, *t*‐test *P* = 0.002, respectively).

3D‐FLAIR postcontrast imaging required for LMCE analysis was available for 88 MS patients (58 RRMS, 30 PMS). Seventeen out of 58 RRMS (29.3%) and 14 out of 30 PMS (46.7%) had at least one definite LMCE.

### Associations between sNfL and MRI–derived lesion and brain volumes

**Figure 2:**
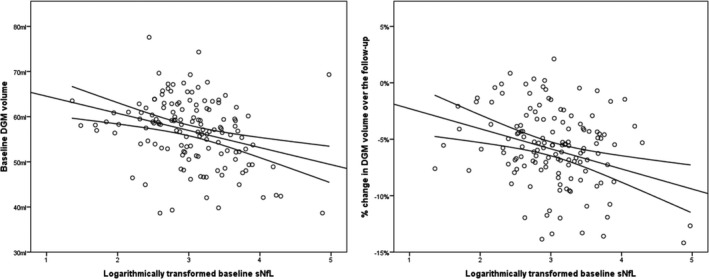
Scatter plot representation of the PwMS associations between baseline sNfL and MRI–derived DGM volume at baseline and the 5‐year longitudinal change. PwMS, persons with multiple sclerosis; DGM, deep gray matter; sNfL, serum neurofilament light chain levels. Due to the data distribution of the baseline sNfL, the value was logarithmically transformed and plotted as such on the *x*‐axis.

Associations between baseline and change of sNfL levels with baseline and change of MRI–derived volumes are shown in Tables 3 and 4. Within the PwMS group, baseline sNfL levels were cross–sectionally associated with T_1_, T_2_ and Gd–enhancing LV (*β* = 0.344, *q* = 0.002; *β* = 0.371, *q* = 0.001; and *β* = 0.508, *q* < 0.001, respectively). Furthermore, higher baseline sNfL levels were associated with lower cross–sectional DGM volume (*β* = −0.257, *q* = 0.017), thalamus (*β* = −0.216, *q* = 0.017), caudate (*β* = −0.263, *q* = 0.014) and hippocampus (*β* = −0.267 *q* = 0.015) volumes. Similarly, the baseline sNfL levels were associated with higher longitudinal loss of the WBV (*β* = −0.356, *q* = 0.002), GMV (*β* = −0.264, *q* = 0.042), and volumes of total DGM (*β* = −0.386, *q* = 0.017; Fig. 2), thalamus (*β* = −0.272, *q* = 0.049), putamen (*β* = −0.395, *q* < 0.001) and globus pallidus (*β* = −0.284, *q* = 0.017). Lastly, PwMS had significant associations between longitudinal increase in sNfL and decrease of GMV (*β* = 0.242, *q* = 0.049) over the follow‐up. The associations between baseline sNfL, cortical atrophy, and longitudinal change in sNfL with concurrent cortical atrophy did not survive multiple comparison correction (*β* = −0.245, *P* = 0.025 and *q* = 0.058) and (*β* = 0.223, *q* = 0.069). In the binary logistic model adjusted for age, sex, baseline DMT use and change in DMT over the follow‐up, baseline sNfL levels were not associated with presence of LMCE at follow‐up (Negelkerke *R* ^2^ = 0.106, *B* = 0.083, S.E.=1.675, *q* = 0.959).

**Table 3:** Associations between sNfL levels and MRI–derived lesion volumes and global brain volumes in HCs and PwMS.

Analysis of sNfL measure and MRI–derived volumes		HCs (*n* = 47)			PwMS (*n* = 120)		
		Baseline MRI	Longitudinal change in MRI		Baseline MRI	Longitudinal change in MRI	
		Baseline sNfL	Baseline sNfL	sNfL change	Baseline sNfL	Baseline sNfL	sNfL change
T_1_‐LV	Standardized *β*	–	–	–	**0.344**	0.005	−0.162
	q‐value	–	–	–	**0.002**	0.426	0.997
T_2_‐LV	Standardized *β*	0.151	0.083	0.028	**0.371**	0.032	−0.005
	*q*‐value	0.963	0.963	0.963	**0.001**	0.962	0.968
Gd‐LV	Standardized *β*	–	–	–	**0.508**	**0.758** *	**1.294** *
	*q*‐value	–	–	–	**<0.001**	**<0.001**	**<0.001**
WBV	Standardized *β*	−0.065	−0.009	−0.096	−0.159	−**0.356**	0.101
	*q*‐value	0.963	0.963	0.963	0.139	**0.002**	0.425
WMV	Standardized *β*	0.221	−0.247	0.024	−0.148	−0.002	−0.1
	*q*‐value	0.963	0.963	0.963	0.227	0.985	0.468
GMV	Standardized *β*	−0.247	0.099	−0.094	−0.102	−**0.264**	**0.242**
	*q*‐value	0.443	0.963	0.963	0.365	**0.042**	**0.049**
CV	Standardized *β*	−0.231	0.129	0.015	−0.123	−0.245	0.223
	*q*‐value	0.379	0.963	0.963	0.253	0.058	0.069

MRI, magnetic resonance imaging; sNfL, serum neurofilament light chain; HCs, healthy controls; PwMS, persons with multiple sclerosis; LV, lesion volume; Gd, gadolinium; WBV, whole brain volume; WMV, white matter volume; GMV, gray matter volume; CV, cortical volume.

Longitudinal change for MRI–lesion derived outcomes was used in mL, whereas for brain volumes, the percentage changes were used.

Regression models using two blocks (block #1 correcting for age, sex, DMT use at baseline, and DMT change over the follow–up period as covariates and block #2 step‐wise addition of sNfL measure) were constructed. The standardized *β* and *P*‐value demonstrate the main effect of sNfL in the model. The *P*‐value from the regression models were corrected for false discovery rate utilizing Benjamini‐Hochberg procedure. *Q*‐values <0.05 were considered significant and displayed in bold.

* The data distribution of the % change in Gd‐LV was normalized with zero‐inflated transformation. Poisson loglinear generalized statistical model was used, where exp(B) values and Benjamini‐Hochberg–corrected q‐values are reported.

**Table 4:** Associations between serum neurofilament light chain levels and MRI–derived deep gray matter volumes in HCs and PwMS.

Analysis of sNfL measure and MRI–derived DGM volumes		HCs (*n* = 47)			PwMS (*n* = 120)		
		Baseline MRI	Longitudinal change in MRI		Baseline MRI	Longitudinal change in MRI	
		Baseline sNfL	Baseline sNfL	sNfL change	Baseline sNfL	Baseline sNfL	sNfL change
DGM	Standardized *β*	0.026	−0.108	−0.261	−**0.257**	−**0.386**	0.121
	*q*‐value	0.963	0.963	0.553	**0.017**	**<0.001**	0.319
Thalamus	Standardized *β*	0.04	−0.085	−0.291	−**0.216**	−**0.272**	0.006
	*q*‐value	0.963	0.963	0.454	**0.017**	**0.049**	0.950
Caudate	Standardized *β*	−0.075	−0.091	−0.037	−**0.263**	−0.075	0.14
	*q*‐value	0.963	0.963	0.963	**0.014**	0.599	0.247
Putamen	Standardized *β*	0.075	0.039	−0.396	−0.196	−**0.395**	0.168
	*q*‐value	0.963	0.963	0.243	0.086	**<0.001**	0.143
Globus pallidus	Standardized *β*	−0.015	−0.215	−0.037	−0.187	−**0.284**	0.071
	*q*‐value	0.963	0.963	0.963	0.114	**0.017**	0.583
Hippocampus	Standardized *β*	0.065	−0.068	−0.107	−**0.267**	−0.027	0.03
	*q*‐value	0.963	0.963	0.963	**0.015**	0.939	0.925

MRI, magnetic resonance imaging; sNfL, serum neurofilament light chain; DGM, deep gray matter; HCs, healthy controls; PwMS, persons with multiple sclerosis.

Percentage longitudinal change was used for DGM outcomes.

Regression models using two blocks (block #1 correcting for age, sex, DMT use at baseline, and DMT change over the follow–up period as covariates and block #2 step‐wise addition of sNfL measure) were constructed. The standardized *β* and *P*‐value demonstrate the main effect of sNfL in the model. The *P*‐value from the regression models were corrected for false discovery rate using Benjamini‐Hochberg procedure. *Q*‐values <0.05 were considered significant and displayed in bold.

There was no association between baseline sNfL, longitudinal sNfL change, and any MRI‐derived volumes within the HCs population (Tables 3 and 4).

The associations between sNfL levels and the MRI–derived volumes were additionally examined within the RRMS and PMS groups separately and are shown in Tables S1 and S2. The aforementioned MS findings were driven mostly by associations seen in the RRMS. RRMS baseline sNfL levels were associated with baseline T_1_‐, T_2_‐ and Gd‐LV (*β* = 0.381, *q* = 0.007), (*β* = 0.402, *q* = 0.003) and (*β* = 0.539, *q* < 0.001) respectively. Baseline sNfL levels were associated also with 5‐year loss of WBV (*β* = −0.378, *q* = 0.005), and volumes of total DGM (*β* = −0.431, *q* = 0.001), thalamus (*β* = −0.331, *q* = 0.021), putamen (*β* = −0.415, *q* = 0.002) and globus pallidus (*β* = −0.296, *q* = 0.042). Furthermore, the longitudinal increase in sNfL was associated with longitudinal GMV (*β* = 0.319, *q* = 0.041). As mentioned previously, both longitudinal change in sNfL and concurrent cortical atrophy association did not survive multiple comparison correction in RRMS as well (*β* = 0.296, *P* = 0.016, *q* = 0.059) Contrarily, the PMS only showed longitudinal associations between baseline sNfL and follow‐up Gd‐LV (exp(B)=0.829, *q* = 0.013) and their concurrent change (exp(B)=1.326, *q* < 0.001).

No associations between sNfL levels and presence of LMCE were found in neither RRMS nor PMS (Nagelkerke *R* ^2^ = 0.042, *q* = 0.833; and Nagelkerke *R* ^2^ = 0.049, *q* = 0.692, respectively).

**Figure 3:**
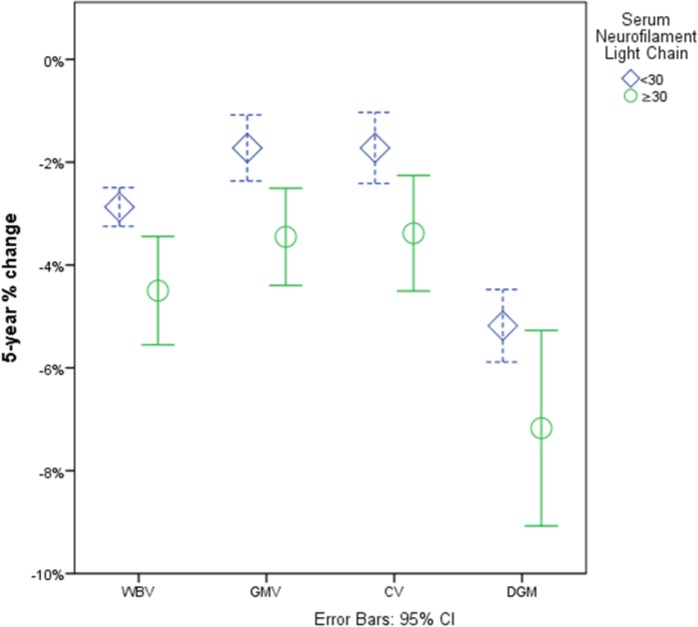
Visual representation of the differences in 5‐year atrophy rate between PwMS with sNfL <30 and ≥30 pg/mL. PwMS, persons with multiple sclerosis; sNfL, serum neurofilament light chain; WBV, whole brain volume; GMV, gray matter volume; CV, cortical volume; DGM, deep gray matter.

In the post hoc analysis, the PwMS were classified based on baseline sNfL of <30 and ≥30 pg/mL (Table 5). The ≥30 pg/mL group was significantly older (52.7 vs. 46.4 years old, *t*‐test *P* = 0.005) and had longer disease duration (20.4 vs. 14.5 years, *t*‐test *P* = 0.005) when compared to the <30 pg/mL group. A numerically larger portion of PwMS ≥30 pg/mL had presence of LMCE (*χ* ^2^‐test, 50.0% vs. 29.7%, *P* = 0.076). Even after ANCOVA correction for age, sex, disease phenotype and EDSS scores, the ≥30 pg/mL group had larger T_2_‐LV (27.0 vs. 11.6 mL, *P* < 0.002) and smaller cross–sectional volumes of total DGM (51.6 vs. 57.5 mL, *P* = 0.0013), thalamus (17.1 vs. 19.0 mL, *P* = 0.046), caudate (7.4 vs. 8.6 mL, *P* = 0.001), putamen (10.9 vs. 12.2 mL, *P* = 0.03) and hippocampus (8.2 vs. 9.2 mL, *P* = 0.009). The ≥30 pg/mL group had greater longitudinal loss of WBV (−4.5% vs. −3.0%, *P* < 0.001), GMV (−3.5% vs. −1.7%, *P* = 0.01), and CV (−3.4% vs. −1.7%, *P* = 0.01) (Fig. 3) In terms of DGM structures, the ≥30 pg/mL group had greater longitudinal volume loss of the thalamus (−6.9% vs. −5.7%, *P* = 0.044) and putamen (−7.9% vs. −3.5, *P* = 0.008).

**Table 5:** Cross–sectional and longitudinal MRI changes between PwMS with <30 and ≥30 pg/mL sNfL levels.

	PwMS (*n* = 120)		
	sNfL < 30 (*n* = 87)	sNfL ≥ 30 (*n* = 33)	*P*‐value
Female, *n* (%)	61 (70.1)	24 (72.7)	0.779
Age, mean (SD)	46.4 (10.8)	52.7 (10.9)	**0.005**
Disease duration, mean (SD)	14.5 (9.8)	20.4 (10.7)	**0.005**
RRMS/PMS	67/20	17/16	**0.007**
EDSS at baseline, median (IQR)	2.5 (2.0–4.0)	6.0 (3.0–6.5)	**<0.001**
LMCE at follow‐up, *n* (%)	19 (29.7)	12 (50.0)	0.076
T_1_‐LV, mean (SD)	2.5 (5.8)	5.9 (11.8)	0.279
T_2_‐LV, mean (SD)	11.6 (14.7)	27.0 (27.1)	**0.002**
Gd‐LV, mean (SD)	0.01 (0.07)	0.3 (1.0)	**<0.001**
WBV, mean (SD)	1466.6 (93.8)	1396.2 (93.8)	**0.131**
WMV, mean (SD)	727.7 (64.4)	694.4 (57.5)	**0.103**
GMV, mean (SD)	738.9 (61.1)	701.7 (62.9)	0.595
CV, mean (SD)	599.4 (48.8)	567.5 (52.2)	0.494
Total DGM, mean (SD)	57.5 (6.9)	51.6 (6.6)	**0.013**
Thalamus, mean (SD)	19.0 (2.5)	17.1 (2.5)	**0.046**
Caudate, mean (SD)	8.6 (1.2)	7.4 (1.3)	**0.001**
Putamen, mean (SD)	12.2 (1.7)	10.9 (1.6)	**0.03**
Globus pallidus, mean (SD)	4.3 (0.7)	4.0 (0.7)	0.369
Hippocampus, mean (SD)	9.2 (1.3)	8.2 (1.1)	**0.009**
Longitudinal change			
T_1_‐LV, mean (SD)	−0.04 (1.1)	1.0 (2.7)	0.898
T_2_‐LV, mean (SD)	0.2 (2.9)	1.1 (8.6)	0.396
Gd‐LV, mean (SD)	−0.009 (0.07)	−0.3 (1.1)	0.054
WBV, mean (SD)	−3.0 (1.8)	−4.5 (2.5)	**<0.001**
WMV, mean (SD)	−1.7 (4.8)	−1.1 (2.6)	0.743
GMV, mean (SD)	−1.7 (2.8)	−3.5 (2.4)	**0.01**
CV, mean (SD)	−1.7 (2.9)	−3.4 (2.8)	**0.01**
Total DGM, mean (SD)	−5.6 (3.5)	−7.1 (4.8)	0.078
Thalamus, mean (SD)	−5.7 (4.4)	−6.9 (4.4)	**0.044**
Caudate, mean (SD)	−5.2 (5.3)	−3.7 (10.7)	0.281
Putamen, mean (SD)	−3.5 (6.6)	−7.9 (8.4)	**0.008**
Globus pallidus, mean (SD)	−9.1 (8.4)	−12.0 (8.9)	0.138
Hippocampus, mean (SD)	−5.7 (7.0)	−4.7 (7.1)	0.337

PwMS, persons with multiple sclerosis; RRMS, relapsing–remitting multiple sclerosis; PMS, progressive multiple sclerosis; DGM, deep gray matter; LMCE, leptomeningeal contrast enhancement; LV, lesion volume; Gd, gadolinium; WBV, whole brain volume; WMV, white matter volume; GMV, gray matter volume; CV, cortical volume; IQR, interquartile range; SD, standard deviation.

Longitudinal change for MRI–lesion derived outcomes was used in mL, whereas for brain volumes, the percentage changes were used.

3D FLAIR postcontrast imaging was available for 64 MS patients <30 pg/mL sNfL and 24 MS patients with ≥30 pg/mL sNfL.

*P*‐values <0.05 were considered significant. Student’s *t*‐test, Mann–Whitney *U*‐test and *χ* ^2^‐test were used appropriately. The MRI–derived comparison between the <30 pg/mL and ≥30 pg/mL groups were derived using analysis of covariance (ANCOVA) adjusted for baseline differences in age, sex, disease phenotype and EDSS scores. In bold are displayed significant *P*‐values.

Based on the age–specific 97.5th and 99th percentiles HC sNfL levels, PwMS were classified with “elevated” sNfL levels, respectively. Twelve (12) PwMS had elevated baseline sNfL above the 97.5th percentile (44.1 pg/mL). There were no differences in age or sex between PwMS with elevated sNfL and the remaining 108 PwMS with baseline sNfL <44.1 pg/mL, but had more PMS patients (*P* = 0.042) and were more disabled at baseline (*P* = 0.004). After utilizing ANCOVA corrected for age, sex, disease phenotype, and baseline EDSS, the PwMS with baseline sNfL ≥44.1 pg/mL had greater future loss of the WBV (−5.5% vs. 3.3%, *P* = 0.003), GMV (−4.9% vs. 1.9%, *P* = 0.01), CV (−5.0% vs. −1.0%, *P* = 0.012), total DGM (−8.9% vs. −5.8%, *P* = 0.033), and putamen (−11.6% vs. −3.8%, *P* = 0.004). Similar findings were found based on the 99th percentile cut‐off (51.9 pg/mL), where the six PwMS with elevated sNfL had greater adjusted longitudinal atrophy of the GMV (*P* = 0.043), and putamen (*P* = 0.013).

Similarly, based on the individual age–specific classification, 12 PwMS were classified with “elevated sNfL levels” by 95th and six PwMS by 97.5th percentile cut‐off. The group with “elevated sNfL levels over 95th percentile” was significantly younger than the group with “normal sNfL levels” (40.9 vs. 48.9 years, *P* = 0.017). Even after adjusting for age, the PwMS with “elevated sNfL levels” had significantly smaller baseline WBV (1413.7 vs. 1450.7 mL, *P* = 0.007), WMV (687.1 vs. 721.9 mL, *P* = 0.018), and all DGM structures including total DGM volume (50.9 vs. 56.5 mL, *P* < 0.001), thalamus (16.6 vs. 18.7 mL, *P* < 0.001), caudate (7.3 vs. 8.4 mL, *P* < 0.001), putamen (11.1 vs. 11.9 mL, *P* = 0.016), globus pallidus (3.9 mL vs. 4.3, *P* = 0.044) and hippocampus (8.2 vs. 9.0 mL, *P* = 0.002). Furthermore, these PwMS had greater baseline T1 and T2 and Gd‐LV (*P* = 0.001, *P* = 0.032, and *P* < 0.001, respectively). Lastly, the individually classified PwMS with “elevated sNfL levels” had greater longitudinal atrophy in WBV (−5.3% vs. −3.2%, *P* = 0.003), GMV (−4.3% vs. −1.9%, *P* = 0.016), CV (−4.4% vs. −1.9%, *P* = 0.029), total DGM (−9.3% vs. −5.7%, *P* = 0.007), and putamen (−10.9% vs. −3.9%, *P* = 0.003). Similarly, PwMS with elevated levels according to the 97.5th percentile individual classification had greater atrophy of the WBV (*P* < 0.001), GM (*P* = 0.004), CV (*P* = 0.015), total DGM (*P* < 0.001), thalamus (*P* = 0.005), putamen (*P* < 0.001), and globus pallidus (*P* = 0.037).

Associations between sNfL levels and MRI–derived brain volumes were further corrected for the extent of active inflammatory activity including Gd–enhancing LV, longitudinal accrual of T1‐LV and of new/enlarging T2‐LV (Table S3). Baseline sNfL levels were associated with baseline hippocampus volume (*β* = −0.246, *P* = 0.024). Moreover, baseline sNfL levels were associated with longitudinal 5‐year change of the WBV (*β* = −0.332, *P* = 0.004), total DGM (*β* = −0.358, *P* = 0.006), thalamus (*β* = −0.28, *P* = 0.013), and putamen (*β* = −0.3, *P* = 0.008).

## Discussion

This study demonstrates cross–sectional and mid‐term longitudinal associations of sNfL levels and concurrent global, cortical, and DGM neurodegenerative pathology in a heterogeneous population of PwMS. More importantly, baseline sNfL levels were associated with the future longitudinal atrophy rate of WBV, GMV, total DGM, thalamus, putamen and globus pallidus. These findings remained significant despite correcting for the extent of baseline inflammatory activity, accrual of new/enlarging T2‐LV and formation of new T1 lesions. Despite the significantly higher sNfL levels seen in the PMS group, the associations between sNfL and the neurodegenerative pathology were mostly driven by the RRMS patients. Lastly, PwMS with sNfL ≥30 pg/mL had lower cross–sectional volumes and greater longitudinal atrophy rate of the global and regional volumes compared to PwMS with sNfL <30 pg/mL. Greater future neurodegenerative pathology was seen within PwMS with baseline sNfLs larger than previously determined age–specific 97.5th and 99th percentile HC sNfL levels and in analysis derived on a patient‐specific level.

Axonal degeneration has been implicated as an important driver of disability accumulation.^29^ More so, the initial demyelination of the axons promotes inefficient energy usage, mitochondrial dysfunction, and accumulation of oxidized stress.^30^ These changes promote axonal fragmentation and ultimately lead to neuronal damage.^30^ The retrograde, transaxonal spread of pathology has been corroborated by the neurodegeneration seen in anatomically distinct tracts and their corresponding GM structures.^6^, ^31^ As the events of axonal retraction are not simultaneous and occur over a certain follow–up period, baseline sNfL levels are expected to be better correlated with later occurring brain atrophy^32^

Our findings of higher baseline sNfL levels, higher concurrent inflammatory activity, and future WBV atrophy are in line with several previous reports.^17^, ^33^, ^34^ Associations between baseline sNfL levels, T_2_‐LV, and Gd–enhancing lesions have been demonstrated as early as at the first demyelinating event, and are able to significantly predict conversion to clinically definite MS.^34^ Moreover, studies report up to 17.8% and 4.9% increase in sNfL levels for every Gd–enhancing lesion and new/enlarging T_2_ lesion, respectively.^17^ In a 10‐year follow‐up study, sNfL levels measured at the 5‐year time point were associated with greater WBV loss till year 10.^33^ Correspondingly, a 5‐year longitudinal study showed similar sNfL effect size and associations with greater WBV atrophy measured both at 2 and 5‐year follow‐up.^17^ While we confirm the sNfL and global brain volume findings, we further demonstrate that the aforementioned associations are specifically driven by changes within cortical and deep GM regions. In order to further delineate the potential relationship between the released sNfL and longitudinal atrophy rate, we conducted a post hoc analysis with adjustment for potential lesion–derived influence. Despite the attenuation of the relationship, baseline sNfL remained associated with both longitudinal whole brain and DGM atrophy. Albeit statistical in nature, this analysis potentially isolates the neurodegenerative axonal propagation from acute lesional sNfL release or depicts the process of primary and independent neuronal neurodegeneration.

Both thalamus and putamen have been highlighted as DGM nuclei with the strongest associations between baseline sNfL levels and its longitudinal atrophy rate. Since the thalamus has broad network of afferent and efferent reciprocal connections with cortical and subcortical regions, the implicated axonal dying–back pathology would be the most apparent. Furthermore, these findings are in line with previous results which demonstrated that lesioned tract disruption is associated with specific increase in regional atrophy of the putamen.^35^ Future cortical parcellation of the study population may provide greater insight regarding associations between the axonal transection, sNfL levels, and atrophy of tract–specific deep GM and cortical surface regions.

Although in our analysis globus pallidus had almost twice the atrophy rate when compared to the remaining DGM nuclei, we detected weaker longitudinal associations with the baseline sNfL levels. These differences in atrophy rate and sNfL associations can be explained by the multifactorial pathophysiological mechanisms of MS neurodegeneration. For example, a recent prospective study showed that globus pallidus and its higher iron–based magnetic susceptibility signal is highly associated with greater physical disability.^36^ Therefore, the differential atrophy of the globus pallidus may be also driven by the processes of iron accumulation and oxidative stress, whereas the atrophy rate of the putamen may be mainly driven by the lesioned neuro–axonal transection and captured by sNfL levels.^37^, ^38^, ^39^ On the other hand, the differential sNfL associations between the RRMS and PMS groups can be interpreted by recent evidence of temporal– and spatial–specific GM atrophy sequences, by a meningeal–driven neurodegeneration and by the relative lack of active inflammation within the aging PMS patients.^40^, ^41^ Certain DGM structures like the thalamus and the caudate have been shown to atrophy early on in the disease, whereas the long‐standing MS disease is associated with more cortical‐oriented atrophy.^41^ Furthermore, PMS patients commonly present with a higher proportion of meningeal infiltrates, the tertiary follicle–like structures which provide compartmentalization of the inflammatory process. In our subanalysis, we were not able to show any cross‐sectional or longitudinal associations between sNfL levels and presence of leptomeningeal pathology, as detected using LMCE on MRI. The discrepancy can be explained by the differential disruption of the brain–blood barrier (BBB) seen in the RRMS and PMS patients.^42^ In addition to the lower overall inflammatory activity, the BBB is relatively intact in PMS patients, which will limit the extravasation of free NfL into the serum.

Admittedly, the 5‐year follow–up study design may have contributed to certain limitations in our interpretation of the biological processes. The two time–point sampling may have been too far apart to estimate the temporal relationship between the sNfL levels and the DGM and cortical atrophy. In addition to the volumetric imaging provided in this study, use of diffusion–tensor imaging or susceptibility–based imaging would provide further understanding of the multiple overlapping neurodegenerative processes. A larger PMS sample size with better characterization of both the inflammatory (detection of cortical lesions) and the neurodegenerative changes can provide better understanding of the sNfL utility in this particular phenotype. Furthermore, to determine the differential DMT effect on the sNfL levels and the progression of MRI brain volume outcomes is of particular clinical importance, and should be further explored. The presence of cardiovascular or other comorbid etiologies may further influence the associations between the sNfL levels and the long–term clinical and MRI–derived outcomes. Lastly, determining age–specific HC sNfL levels derived from large databases would allow better classification of abnormal sNfL levels within the increasingly older MS populations. 43

In conclusion, the sNfL levels measured in a heterogeneous MS population are associated with higher inflammatory and future neurodegenerative pathology. The sNfL measurement is a convenient and easily accessible tool in determining MS patients who are at higher neurodegenerative risk.

## Author’s Contribution

Dejan Jakimovski ‐ Study concept and design; analysis and interpretation; critical revision of the manuscript for important intellectual content; study supervision. Jens Kuhle ‐ Study concept and design; analysis and interpretation; critical revision of the manuscript for important intellectual content. Christian Barro ‐ Study concept and design; analysis and interpretation; critical revision of the manuscript for important intellectual content. Murali Ramanathan ‐ Study concept and design; analysis and interpretation; critical revision of the manuscript for important intellectual content; study supervision. Jesper Hagemeier ‐ Analysis and interpretation; critical revision of the manuscript for important intellectual content, Niels Bergsland ‐ Analysis and interpretation; critical revision of the manuscript for important intellectual content. Davorka Tomic ‐ Critical revision of the manuscript for important intellectual content. Harald Kropshofer ‐ Critical revision of the manuscript for important intellectual content. David Leppert ‐ Critical revision of the manuscript for important intellectual content. Zuzanna Michalak ‐ Analysis and interpretation; critical revision of the manuscript for important intellectual content. Michael G. Dwyer ‐ Analysis and interpretation; critical revision of the manuscript for important intellectual content. Ralph HB Benedict ‐ Study concept and design; analysis and interpretation; critical revision of the manuscript for important intellectual content; study supervision. Bianca Weinstock‐Guttman ‐ Study concept and design; analysis and interpretation; critical revision of the manuscript for important intellectual content; study supervision. Robert Zivadinov ‐ Study concept and design; analysis and interpretation; critical revision of the manuscript for important intellectual content; study supervision.

## Conflict of Interest

Dejan Jakimovski, Jesper Hagemeier, Niels Bergsland and Zuzanna Michalak have nothing to disclose.

Jens Kuhle received speaker fees, research support, travel support, and/or served on advisory boards by ECTRIMS, Swiss MS Society, Swiss National Research Foundation, (320030_160221), University of Basel, Bayer, Biogen, Genzyme, Merck, Novartis, Protagen AG, Roche, Teva.

Christian Barro received conference travel grant from Teva and Novartis.

Murali Ramanathan received research funding or consulting fees from the National Multiple Sclerosis Society, the Department of Defense, the National Institutes of Health, National Science Foundation and Otuska Pharmaceutical Development.

Davorka Tomic, Harald Kropshofer and David Leppert are employees of Novartis Pharma AG, Basel, Switzerland.

Michael G. Dwyer has received consultant fees from Claret Medical and EMD Serono.

Ralph RH. Benedict received personal compensation from Neurocog Trials, Genentech, Roche, Takeda, Abbvie, Novartis, Sanofi and EMD Serono for speaking and consultant fees. He received financial support for research activities from Genzyme, Biogen, Mallinckrodt.

Bianca Weinstock‐ Guttman received honoraria as a speaker and as a consultant for Biogen Idec, EMD Serono, Genentech, Novartis and Mallinckrodt. Dr. Weinstock‐Guttman received research funds from Biogen Idec, Genentech, EMD Serono, and Novartis.

Robert Zivadinov received personal compensation from EMD Serono, Genzyme‐Sanofi, Celgene and Novartis for speaking and consultant fees. He received financial support for research activities from Genzyme‐Sanofi, Novartis, Celgene, Mapi Pharma and Protembis.

## Supporting information


**Table S1.** Associations between sNfL and MRI‐derived lesion and global brain volumes in RRMS and PMS subpopulations.
**Table S2.** Associations between sNfL and MRI–derived DGM volumes in RRMS and PMS subpopulations.
**Table S3.** Associations between sNfL levels and MRI–derived brain volumes in MS patients after correcting for inflammatory activity including baseline gadolinium lesion volume, accrual of T1‐ lesion volume and new and enlarging T2‐ lesion volume.

**Data S1** **.** MRI acquisition parameters.

## References

[1] Benedict RH , Zivadinov R . Risk factors for and management of cognitive dysfunction in multiple sclerosis. Nat Rev Neurol 2011;7:332–342. 21556031 10.1038/nrneurol.2011.61

[2] Fisher E , Lee JC , Nakamura K , Rudick RA . Gray matter atrophy in multiple sclerosis: a longitudinal study. Ann Neurol 2008;64:255–265. 18661561 10.1002/ana.21436

[3] Zivadinov R , Jakimovski D , Gandhi S , et al. Clinical relevance of brain atrophy assessment in multiple sclerosis. Implications for its use in a clinical routine. Expert Rev Neurother 2016;16:777–793. 27105209 10.1080/14737175.2016.1181543

[4] Comabella M , Montalban X . Body fluid biomarkers in multiple sclerosis. Lancet Neurol 2014;13:113–126. 24331797 10.1016/S1474-4422(13)70233-3

[5] Friese MA , Schattling B , Fugger L . Mechanisms of neurodegeneration and axonal dysfunction in multiple sclerosis. Nat Rev Neurol 2014;10:225–238. 24638138 10.1038/nrneurol.2014.37

[6] Kolasinski J , Stagg CJ , Chance SA , et al. A combined post‐mortem magnetic resonance imaging and quantitative histological study of multiple sclerosis pathology. Brain 2012;135(Pt 10):2938–2951. 23065787 10.1093/brain/aws242 PMC3470716

[7] Geurts JJ , Calabrese M , Fisher E , Rudick RA . Measurement and clinical effect of grey matter pathology in multiple sclerosis. Lancet Neurol 2012;11:1082–1092. 23153407 10.1016/S1474-4422(12)70230-2

[8] Jakimovski D , Weinstock‐Guttman B , Hagemeier J , et al. Walking disability measures in multiple sclerosis patients: correlations with MRI‐derived global and microstructural damage. J Neurol Sci 2018;393:128–134. 30165291 10.1016/j.jns.2018.08.020

[9] Eshaghi A , Prados F , Brownlee WJ , et al. Deep gray matter volume loss drives disability worsening in multiple sclerosis. Ann Neurol 2018;83:210–222. 29331092 10.1002/ana.25145 PMC5838522

[10] Zivadinov R , Havrdova E , Bergsland N , et al. Thalamic atrophy is associated with development of clinically definite multiple sclerosis. Radiology 2013;268:831–841. 23613615 10.1148/radiol.13122424

[11] Azevedo CJ , Cen SY , Khadka S , et al. Thalamic atrophy in multiple sclerosis: a magnetic resonance imaging marker of neurodegeneration throughout disease. Ann Neurol 2018;83:223–234. 29328531 10.1002/ana.25150 PMC6317847

[12] Magliozzi R , Howell OW , Reeves C , et al. A gradient of neuronal loss and meningeal inflammation in multiple sclerosis. Ann Neurol 2010;68:477–493. 20976767 10.1002/ana.22230

[13] Zivadinov R , Ramasamy DP , Vaneckova M , et al. Leptomeningeal contrast enhancement is associated with progression of cortical atrophy in MS: a retrospective, pilot, observational longitudinal study. Mult Scler 2017;23:1336–1345. 27811339 10.1177/1352458516678083

[14] Khalil M , Teunissen CE , Otto M , et al. Neurofilaments as biomarkers in neurological disorders. Nat Rev Neurol 2018;14:577–589. 30171200 10.1038/s41582-018-0058-z

[15] Kuhle J , Barro C , Andreasson U , et al. Comparison of three analytical platforms for quantification of the neurofilament light chain in blood samples: ELISA, electrochemiluminescence immunoassay and Simoa. Clin Chem Lab Med 2016;54:1655–1661. 27071153 10.1515/cclm-2015-1195

[16] Disanto G , Barro C , Benkert P , et al. Serum neurofilament light: a biomarker of neuronal damage in multiple sclerosis. Ann Neurol 2017;81:857–870. 28512753 10.1002/ana.24954 PMC5519945

[17] Barro C , Benkert P , Disanto G , et al. Serum neurofilament as a predictor of disease worsening and brain and spinal cord atrophy in multiple sclerosis. Brain 2018;141:2382–2391. 29860296 10.1093/brain/awy154

[18] Jakimovski D , Gandhi S , Paunkoski I , et al. Hypertension and heart disease are associated with development of brain atrophy in multiple sclerosis: a 5‐year longitudinal study. Eur J Neurol 2019;26:87‐e8. 30103277 10.1111/ene.13769

[19] Polman CH , Reingold SC , Banwell B , et al. Diagnostic criteria for multiple sclerosis: 2010 revisions to the McDonald criteria. Ann Neurol 2011;69:292–302. 21387374 10.1002/ana.22366 PMC3084507

[20] Kurtzke JF . Rating neurologic impairment in multiple sclerosis: an expanded disability status scale (EDSS). Neurology 1983;33:1444–1452. 6685237 10.1212/wnl.33.11.1444

[21] Zivadinov R , Heininen‐Brown M , Schirda CV , et al. Abnormal subcortical deep‐gray matter susceptibility‐weighted imaging filtered phase measurements in patients with multiple sclerosis: a case‐control study. NeuroImage 2012;59:331–339. 21820063 10.1016/j.neuroimage.2011.07.045

[22] Dwyer MG , Bergsland N , Ramasamy DP , et al. Atrophied brain lesion volume: a new imaging biomarker in multiple sclerosis. J Neuroimaging 2018;28:490–495. 29856910 10.1111/jon.12527

[23] Gelineau‐Morel R , Tomassini V , Jenkinson M , et al. The effect of hypointense white matter lesions on automated gray matter segmentation in multiple sclerosis. Hum Brain Mapp 2012;33:2802–2814. 21976406 10.1002/hbm.21402 PMC5435105

[24] Smith KJ , Lassmann H . The role of nitric oxide in multiple sclerosis. Lancet Neurol 2002;1:232–241. 12849456 10.1016/s1474-4422(02)00102-3

[25] Dwyer MG , Bergsland N , Zivadinov R . Improved longitudinal gray and white matter atrophy assessment via application of a 4‐dimensional hidden Markov random field model. NeuroImage 2014;90:207–217. 24333394 10.1016/j.neuroimage.2013.12.004

[26] Patenaude B , Smith SM , Kennedy DN , Jenkinson M . A Bayesian model of shape and appearance for subcortical brain segmentation. NeuroImage 2011;56:907–922. 21352927 10.1016/j.neuroimage.2011.02.046 PMC3417233

[27] Absinta M , Vuolo L , Rao A , et al. Gadolinium‐based MRI characterization of leptomeningeal inflammation in multiple sclerosis. Neurology 2015;85:18–28. 25888557 10.1212/WNL.0000000000001587 PMC4501940

[28] Kuhle J , Kropshofer H , Haering D , et al. Blood neurofilament light chain as a biomarker of MS disease activity and treatment response. Neurology 2019;92 DOI: 10.1212/WNL.0000000000007032 PMC6442011 30737333

[29] Mahad DH , Trapp BD , Lassmann H . Pathological mechanisms in progressive multiple sclerosis. Lancet Neurol 2015;14:183–193. 25772897 10.1016/S1474-4422(14)70256-X

[30] Fischer MT , Wimmer I , Hoftberger R , et al. Disease‐specific molecular events in cortical multiple sclerosis lesions. Brain 2013;136(Pt 6):1799–815. 23687122 10.1093/brain/awt110 PMC3673462

[31] Bergsland N , Tavazzi E , Lagana MM , et al. White matter tract injury is associated with deep gray matter iron deposition in multiple sclerosis. J Neuroimaging 2017;27:107–113. 27239049 10.1111/jon.12364

[32] Trapp BD , Peterson J , Ransohoff RM , et al. Axonal transection in the lesions of multiple sclerosis. N Engl J Med 1998;338:278–285. 9445407 10.1056/NEJM199801293380502

[33] Chitnis T , Gonzalez C , Healy BC , et al. Neurofilament light chain serum levels correlate with 10‐year MRI outcomes in multiple sclerosis. Ann Clin Transl Neurol 2018;5:1478–1491. 30564615 10.1002/acn3.638 PMC6292183

[34] Dalla Costa G , Martinelli V , Sangalli F , et al. Prognostic value of serum neurofilaments in patients with clinically isolated syndromes. Neurology 2019;92:e733–e741. 30635483 10.1212/WNL.0000000000006902 PMC6382362

[35] Fuchs TA , Carolus K , Benedict RHB , et al. Impact of focal white matter damage on localized subcortical gray matter atrophy in multiple sclerosis: a 5‐year study. Am J Neuroradiol 2018;39:1480–1486. 29976833 10.3174/ajnr.A5720 PMC7410563

[36] Zivadinov R , Tavazzi E , Bergsland N , et al. Brain iron at quantitative MRI is associated with disability in multiple sclerosis. Radiology 2018;289:487–496. 30015589 10.1148/radiol.2018180136 PMC6219694

[37] Haider L , Zrzavy T , Hametner S , et al. The topograpy of demyelination and neurodegeneration in the multiple sclerosis brain. Brain 2016;139(Pt 3):807–815. 26912645 10.1093/brain/awv398 PMC4766379

[38] Schweser F , Raffaini Duarte Martins AL , Hagemeier J , et al. Mapping of thalamic magnetic susceptibility in multiple sclerosis indicates decreasing iron with disease duration: A proposed mechanistic relationship between inflammation and oligodendrocyte vitality. NeuroImage 2018;167:438–452. 29097315 10.1016/j.neuroimage.2017.10.063 PMC5845810

[39] Stephenson E , Nathoo N , Mahjoub Y , et al. Iron in multiple sclerosis: roles in neurodegeneration and repair. Nat Rev Neurol 2014;10:459–468. 25002107 10.1038/nrneurol.2014.118

[40] Steenwijk MD , Geurts JJ , Daams M , et al. Cortical atrophy patterns in multiple sclerosis are non‐random and clinically relevant. Brain 2016;139(Pt 1):115–126. 26637488 10.1093/brain/awv337

[41] Eshaghi A , Marinescu RV , Young AL , et al. Progression of regional grey matter atrophy in multiple sclerosis. Brain 2018;141:1665–1677. 29741648 10.1093/brain/awy088 PMC5995197

[42] Correale J , Gaitan MI , Ysrraelit MC , Fiol MP . Progressive multiple sclerosis: from pathogenic mechanisms to treatment. Brain 2017;140:527–546. 27794524 10.1093/brain/aww258

[43] Vaughn CB , Jakimovski D , Kavak KS , et al. Epidemiology and treatment of multiple sclerosis in elderly populations. Nat Rev Neurol 2019;15:329–342. 31000816 10.1038/s41582-019-0183-3

