# Supplementary material for: Serum neurofilament light chain levels associations with gray matter pathology: a 5‐year longitudinal study
Source: Ann Clin Transl Neurol. 2019 Aug 22;6(9):1757–70. doi: 10.1002/acn3.50872 (PMC6764487; doi:10.1002/acn3.50872)
Supplement: Supplementary file 1 — Table S1. Associations between sNfL and MRI‐derived lesion and global brain volumes in RRMS and PMS subpopulations. Table S2. Associations between sNfL and MRI–derived DGM volumes in RRMS and PMS subpopulations. Table S3. Associations between sNfL levels and MRI–derived brain volumes in MS patients after correcting for inflammatory activity including baseline gadolinium lesion volume, accrual of T1‐ lesion volume and new and enlarging T2‐ lesion volume. [file ACN3-6-1757-s001.docx]

**Supplement Table 1.** Associations between sNfL and MRI-derived lesioned and global brain volumes in RRMS and PMS subpopulations.

| Analysis of sNfL measure in relation with MRI-derived lesion volumes and global brain volumes | | RRMS (n=84) | | | PMS (n=36) | | |
| --- | --- | --- | --- | --- | --- | --- | --- |
|  |  | Baseline MRI | Longitudinal change in MRI | | Baseline MRI | Longitudinal change in MRI | |
|  |  | Baseline sNfL | Baseline sNfL | sNfL change | Baseline sNfL | Baseline sNfL | sNfL change |
| T_1_-LV | Standardized β | **0.381** | 0.143 | -0.388 | 0.171 | 0.338 | -0.199 |
|  | q-value | **0.007** | 0.607 | 0.207 | 0.852 | 0.418 | 0.748 |
| T_2_-LV | Standardized β | **0.383** | 0.097 | 0.001 | 0.182 | -0.203 | 0.222 |
|  | q-value | **0.003** | 0.64 | 0.997 | 0.831 | 0.771 | 0.748 |
| Gd-LV | Standardized β | **0.539** | **0.753*** | **1.293*** | 0.4 | **0.829*** | **1.326*** |
|  | q-value | **<0.001** | **<0.001** | **<0.001** | 0.318 | **0.013** | **<0.001** |
| WBV | Standardized β | -0.111 | **-0.378** | 0.107 | -0.289 | -0.148 | -0.038 |
|  | q-value | 0.493 | **0.005** | 0.481 | 0.412 | 0.747 | 0.958 |
| WMV | Standardized β | -0.091 | 0.0 | -0.153 | -0.141 | -0.228 | 0.041 |
|  | q-value | 0.588 | 1.0 | 0.363 | 0.793 | 0.944 | 0.943 |
| GMV | Standardized β | -0.07 | -0.267 | **0.319** | -0.388 | 0.086 | -0.206 |
|  | q-value | 0.601 | 0.102 | **0.041** | 0.268 | 0.863 | 0.884 |
| CV | Standardized β | -0.101 | -0.23 | 0.296 | -0.401 | 0.094 | -0.219 |
|  | q-value | 0.495 | 0.169 | 0.059 | 0.222 | 0.849 | 0.885 |

**Legend:** RRMS – relapsing-remitting multiple sclerosis, PMS – progressive multiple sclerosis, sNfL – serum neurofilament light chain, LV – lesion volume, Gd – gadolinium, WBV – whole brain volume, WMV – white matter volume, GMV – gray matter volume, CV – cortical volume.

Longitudinal change for MRI-lesion derived outcomes was absolute change in ml, whereas for brain volumes, the percentage changes were used.

Regression models using two blocks (block #1 correcting for age, sex , DMT use at baseline, and DMT change over the follow-up period as covariates and block #2 step-wise addition of sNfL measure) were constructed. The standardized β and p-value demonstrate the main effect of sNfL in the model. The p-value from the regression models were corrected for false discovery rate using Benjamini-Hochberg procedure. Q-values <0.05 were considered significant and displayed in bold.

*The data distribution of the % change in Gd-LV was normalized with zero-inflated transformation. Poisson loglinear generalized statistical model was used, where exp(B) values and Benjamini-Hochberg-corrected q-values are reported.

**Supplement Table 2.** Associations between sNfL and MRI-derived DGM volumes in RRMS and PMS subpopulations.

| Analysis of sNfL measure and MRI-derived DGM volumes | | RRMS (n=84) | | | PMS (n=36) | | |
| --- | --- | --- | --- | --- | --- | --- | --- |
|  |  | Baseline MRI | Longitudinal change in MRI | | Baseline MRI | Longitudinal change in MRI | |
|  |  | Baseline sNfL | Baseline sNfL | sNfL change | Baseline sNfL | Baseline sNfL | sNfL change |
| DGM | Standardized β | -0.235 | **-0.431** | 0.153 | -0.379 | 0.109 | -0.024 |
|  | q-value | 0.096 | **0.001** | 0.281 | 0.215 | 0.768 | 0.949 |
| Thalamus | Standardized β | -0.191 | **-0.331** | -0.054 | -0.336 | 0.142 | 0.163 |
|  | q-value | 0.215 | **0.021** | 0.691 | 0.295 | 0.792 | 0.859 |
| Caudate | Standardized β | -0.233 | -0.083 | 0.171 | -0.391 | 0.071 | -0.102 |
|  | q-value | 0.096 | 0.612 | 0.253 | 0.188 | 0.852 | 0.806 |
| Putamen | Standardized β | -0.173 | **-0.415** | 0.236 | -0.266 | -0.146 | -0.065 |
|  | q-value | 0.262 | **0.002** | 0.1 | 0.428 | 0.829 | 0.853 |
| Globus pallidus | Standardized β | -0.19 | **-0.296** | 0.093 | -0.156 | 0.023 | -0.344 |
|  | q-value | 0.212 | **0.042** | 0.569 | 0.843 | 0.908 | 0.327 |
| Hippocampus | Standardized β | -0.261 | -0.014 | 0.074 | -0.41 | 0.113 | 0.025 |
|  | q-value | 0.063 | 0.95 | 0.609 | 0.242 | 0.755 | 0.926 |

**Legend:** MRI – magnetic resonance imaging, sNfL – serum neurofilament light chain, DGM – deep gray matter, RRMS – relapsing-remitting multiple sclerosis, PMS – progressive multiple sclerosis.

Percentage longitudinal change was used for DGM outcomes.

Regression models using two blocks (block #1 correcting for age, sex, DMT use at baseline, and DMT change over the follow-up period as covariates and block #2 step-wise addition of sNfL measure) were constructed. The standardized β and p-value demonstrate the main effect of sNfL in the model. The p-value from the regression models were corrected for false discovery rate using Benjamini-Hochberg procedure. Q-values <0.05 were considered significant and displayed in bold.

**Supplement Table 3**. Associations between sNfL levels and MRI-derived brain volumes in MS patients after correcting for inflammatory activity including baseline gadolinium lesion volume, accrual of T1- lesion volume and new and enlarging T2- lesion volume.

| Analysis of sNfL measure and MRI-derived volumes | | PwMS (n=120) | | |
| --- | --- | --- | --- | --- |
|  |  | Baseline MRI | Longitudinal change in MRI | |
|  |  | Baseline sNfL | Baseline sNfL | sNfL change |
| WBV | Standardized β | -0.15 | **-0.332** | 0.063 |
|  | q-value | 0.157 | **0.004** | 0.577 |
| WMV | Standardized β | -0.179 | -0.034 | -0.081 |
|  | q-value | 0.12 | 0.798 | 0.53 |
| GMV | Standardized β | -0.05 | -0.177 | 0.236 |
|  | q-value | 0.63 | 0.159 | 0.052 |
| CV | Standardized β | -0.057 | -0.162 | 0.203 |
|  | q-value | 0.58 | 0.199 | 0.1 |
| DGM | Standardized β | -0.178 | **-0.302** | 0.069 |
|  | q-value | 0.103 | **0.006** | 0.528 |
| Thalamus | Standardized β | -0.148 | **-0.28** | 0.012 |
|  | q-value | 0.183 | **0.013** | 0.912 |
| Caudate | Standardized β | -0.131 | 0.039 | 0.127 |
|  | q-value | 0.221 | 0.742 | 0.274 |
| Putamen | Standardized β | -0.11 | **-0.3** | 0.071 |
|  | q-value | 0.327 | **0.008** | 0.529 |
| Globus pallidus | Standardized β | -0.138 | -0.124 | -0.042 |
|  | q-value | 0.232 | 0.287 | 0.713 |
| Hippocampus | Standardized β | **-0.246** | -0.098 | 0.129 |
|  | q-value | **0.024** | -0.87 | 0.244 |

**Legend:** MRI – magnetic resonance imaging, sNfL – serum neurofilament light chain, PwMS – persons with multiple sclerosis, WBV – whole brain volume, WMV – white matter volume, GMV – gray matter volume, CV – cortical volume.

Longitudinal brain volume change was calculated as percentage change over the follow-up.

Regression models using two blocks (block #1 to correcting for age, sex, DMT use at baseline, DMT change over the follow-up period, baseline Gd-LV, change in T1-LV, in new and enlarging T2-LV as covariates and block #2 step-wise addition of sNfL levels) were constructed. The standardized β and p-value demonstrate the main effect of sNfL in the model. P-values <0.05 were considered significant and displayed in bold.
