# Supplementary material for: Serum neurofilament light chain levels associations with gray matter pathology: a 5‐year longitudinal study
Source: Ann Clin Transl Neurol. 2019 Aug 22;6(9):1757–70. doi: 10.1002/acn3.50872 (PMC6764487; doi:10.1002/acn3.50872)
Supplement: Supplementary file 2 — Data S1 . MRI acquisition parameters. [file ACN3-6-1757-s002.docx]

**MRI acquisition parameters:**

The MRI examinations were performed on 3T GE Signa Excite HD 12 Twin Speed 8-channel scanner (General Electric, Milwaukee, WI, USA) with an 8-channel head and neck (HDNV) coil. The sequences that were utilized in this sub-study included: 1) pre-contrast 3D high resolution T_1_-weighted imaging (WI) with fast, spoiled, gradient echo for magnetization-prepared inversion recovery pulse (IR-FSPGR) with echo time (TE)/inversion time (TI)/repetition time (TR) of 2.8ms/900ms/5.9ms, flip angle (FLIP) of 10°, field of view (FOV) of 25.6cm x 19.2cm, voxel size of 1x1x1mm^3^ with no gaps, for use of brain volume measurements 2) 2D-Fluid-Attenuated Inversion-Recovery (FLAIR) imaging with TE/TI/TR of 120ms/2100ms/8500ms, FLIP of 90°, echo train length of 24, FOV of 25.6cm x 19.2cm, voxel size of 1x1x3 mm^3^ with no gaps, for use of T_2_ lesion measurements 3) pre- and post-contrast (acquired after single 0.1mmol/kg gadobutrol injection) spin-echo (SE) T_1_-WI with sequence parameters of TR/TE of 16ms/600ms, FLIP of 90°, FOV of 25.6cm x 19.2cm, voxel size of 1x1x3 mm^3^, for use of T_1_ lesion pre- and post-contrast measurements, and 4) only available at the follow-up MRI scan, a 3D-FLAIR sequence with TR/TI/TE of 9000ms/2420ms/600ms, FOV of 25.6cm x19.2cm, voxel size of 1x1.3x1.3 mm^3^, acquired after 10 minutes delay of the gadobutrol injection, as previously described, for use of leptomeningeal contrast enhancement (LMCE) assessments. ^1^

**Additional metrics for all regression-derived results reported within the main text of the manuscript:**

Associations between baseline and change of sNfL levels with baseline and change of MRI-derived volumes are shown in Tables 3 and 4 of the main manuscript. Within the PwMS group, baseline sNfL levels were cross-sectionally associated with T_1_, T_2_ and Gd-enhancing LV (R^2^=0.178, B=1.869, S.E.=0.517, β=0.344, q=0.002; R^2^=0.195, B=1,01, S.E.=0.25, β=0.371, q=0.001; and R^2^=0.348, B=1.501, S.E.=0.249, β=0.508, q<0.001, respectively). Furthermore, higher baseline sNfL levels were associated with lower cross-sectional DGM volume (R^2^=0.221, B=-3114,9, S.E.=1106,5, β=-0.257, q=0.017), thalamus (R^2^=0.203, B=956, 7, S.E.=405.6, β=-0.216, q=0.017), caudate (R^2^=0.227, B=-584,4, S.E.=200,1, β=-0.263, q=0.014) and hippocampus (R^2^=0.209, B=-583,8, S.E.=199,7, β=-0.267 q=0.015) volumes. Similarly, the baseline sNfL levels were associated with higher longitudinal loss of the WBV (R^2^=0.174, B=-1,328, S.E.=0.331, β=-0.356, q=0.002), GMV (R^2^=0.09, B=-1.215, S.E.=0.491, β=-0.264, q=0.042), , and volumes of total DGM (R^2^=0.171, B=-2.517, S.E.=0.607, β=-0.386, q=0.017; Figure 2), thalamus (R^2^=0.098, B=-2,023, S.E.=0.721, β=-0.272, q=0.049), putamen (R^2^=0.162, B=-4,887, S.E.=1.157, β=-0.395, q<0.001) and globus pallidus (R^2^=0.096, B=-4.093, S.E.=1,398, β=-0.284, q=0.017). Lastly, PwMS had significant associations between longitudinal increase in sNfL and decrease of GMV (R^2^=0.086, B=1,104, S.E.=0.462, β=0.242, q=0.049) over the follow-up. The associations between baseline sNfL and cortical atrophy and longitudinal change in sNfL with concurrent cortical atrophy did not survive multiple comparison correction (R^2^=0. 075, B=-1,227, S.E.=0.538, β=-0.245, p=0.025 and q=0.058) and (R^2^=0 , B=1,109, S.E.=0.507, β=0.223, q=0.069).

Similarly, the associations between sNfL levels and the MRI-derived volumes were additionally examined within RRMS and PMS groups separately and are shown in Supplement Tables 1 and 2 of the main manuscript. The aforementioned MS findings were driven mostly by associations seen in the RRMS. RRMS baseline sNfL levels were associated with baseline T_1_-, T_2_- and Gd-LV (R^2^=0.177, B=2.02, S.E.=0.603, β=0.381, q=0.007), (R^2^=0.177, B=1,106, S.E.=0.303, β=0.402, q=0.003) and (R^2^=0.375, B=1.736, S.E.=0.313, β=0.539, q<0.001) respectively. Baseline sNfL levels were associated also with 5-year loss of WBV (R^2^=0.241, B=-1.336, S.E.=0.383, β=-0.378, q=0.005), and volumes of total DGM (R^2^=0.246, B=-2.921, S.E.=0.714, β=-0.431, q=0.001), thalamus (R^2^=0.134, B=-2.529, S.E.=0.862, β=-0.331, q=0.021), putamen (R^2^=0.222, B=-5.569, S.E.=1.435, β=-0.415, q=0.002) and globus pallidus (R^2^=0.13, B=-4.586, S.E.=1.757, β=-0.296, q=0.042). Furthermore, the longitudinal increase in sNfL was associated with longitudinal GMV (R^2^=0.148, B=-1.389, S.E.=0.521, β=0.319, q=0.041) As mentioned previously, both longitudinal change in sNfL and concurrent cortical atrophy association did not survive multiple comparison correction in RRMS as well (R^2^=0.136, B=1.399, S.E.=0.57, β=0.296, p=0.016, q=0.059) Contrarily, the PMS only showed longitudinal associations between baseline sNfL and follow-up Gd-LV (exp(B)=0.829, q=0.013) and their concurrent change (exp(B)=1.326, q<0.001).

Lastly, associations between sNfL levels and MRI-derived brain volumes were further corrected for the extent of active inflammatory activity including Gd-enhancing LV, longitudinal accrual of T1-LV and of new/enlarging T2-LV (Supplement Table 3 of the main manuscript). Baseline sNfL levels were associated with baseline hippocampus volume (R^2^=0.221, B=-583.7, S.E.=254,5, β=-0.246, p=0.024). Moreover, baseline sNfL levels were associated with longitudinal 5-year change of the WBV (R^2^=0.2, B=-1.314, S.E.=0.446, β=-0.332, p=0.004), total DGM (R^2^=0.218, B=-2.179, S.E.=0.777, β=-0.358, p=0.006), thalamus (R^2^=0.184, B=-2.297, S.E.=0.903, β=-0.28, p=0.013), and putamen (R^2^=0.164, B=-4,076, S.E.=1.511, β=-0.3, p=0.008).

**Reference:**

1. Polak P, Magnano C, Zivadinov R, Poloni G. 3D FLAIRED: 3D fluid attenuated inversion recovery for enhanced detection of lesions in multiple sclerosis. Magn Reson Med. 2012 Sep;68(3):874-81.
